# Grand challenge: building a digitally enabled one health future for pediatric infectious diseases

**DOI:** 10.3389/fped.2026.1967922

**Published:** 2026-09-01

**Authors:** Daniele Zama

**Affiliations:** 1 Department of Medical and Surgical Sciences, Alma Mater Studiorum, University of Bologna, Bologna, Italy; 2 Pediatric Unit, IRCCS Azienda Ospedaliero-Universitaria di Bologna, Bologna, Italy

**Keywords:** antimicrobial stewardship, artificial intelligence, climate change, digital health, healthcare-associated infections, microbiome, one health, pediatric infectious diseases

## Children at the intersection of connected threats

Pediatric infectious diseases are moving into a phase where the old boundaries of the specialty just don't hold up anymore. A febrile child might be the first visible point in a much longer chain.

In 2024, a dengue outbreak in Fano, Italy, hit 199 locally acquired cases, and France logged 83 autochthonous infections—numbers that would have seemed out of place in continental Europe a decade ago (1). For pediatric infectious disease specialists, these aren't just epidemiological oddities; they show how climate, competent vectors, human mobility, and delayed recognition can all converge fast. But the clinical encounter only helps with earlier outbreak detection, safer prescribing, and prevention if clinical data actually link up with microbiological, entomological, environmental, and public health signals. So the real challenge isn't just responding to more pathogens—it's building a child-centered learning system that connects human, animal, and environmental health through responsible digital transformation.

One Health gives us the conceptual framework for this shift. It starts from the idea that the health of people, animals, plants, and ecosystems is all interdependent, and the Quadripartite One Health Joint Plan of Action flags zoonoses, vector-borne diseases, food safety, antimicrobial resistance (AMR), and environmental health as connected priorities (2, 3). Yet One Health often stays an aspiration rather than a routine part of pediatric practice. Digital transformation could supply the connective tissue: interoperable surveillance, geospatial analysis, pathogen genomics, environmental sensors, electronic health records, and artificial intelligence (AI) can turn fragmented observations into shared situational awareness. Technology, though, isn't the end goal. Its value lies in enabling earlier, more equitable, and more sustainable decisions for children.

## Why pediatric infectious diseases need its own one health lens

Children aren't just small adults, either biologically or in terms of exposure. Immune maturation, age-dependent clinical phenotypes, vaccine schedules, developmental behavior, and weight-based treatment all shape susceptibility and outcomes. Their environments are distinct too: households, schools, childcare facilities, playgrounds, farms, and urban transport determine contact with pathogens, animals, pollutants, and vectors. Infants and young children have higher ventilation relative to body size, frequent hand-to-mouth activity, and little control over their surroundings. Social deprivation, displacement, and unequal access to vaccination, sanitation, diagnostics, and digital infrastructure further concentrate risk.

Climate change makes these interdependencies more visible by the year. Temperature, rainfall, drought, flooding, and ecosystem disruption influence the geography and seasonality of vector-borne, waterborne, foodborne, and respiratory infections. Europe's experience with locally acquired arboviruses shows how competent vectors, favorable weather, and imported index cases can converge to create autochthonous transmission, while pediatric infections may stay underdiagnosed because they're mild or nonspecific (1). Air pollution adds another layer: it can damage epithelial barriers, modify immune responses, and raise the risk or severity of respiratory infections in children (4). Environmental exposures may also reshape the respiratory microbiota and favor colonization by potential pathogens (5). The gut and airway microbiomes, then, should be seen as dynamic interfaces between the child, antimicrobials, nutrition, pathogens, and the environment—not isolated laboratory curiosities.

## Digital transformation as an operating system for one health

Current surveillance systems are usually separated by institution, discipline, and administrative level. Pediatric clinical data sit in hospital records; microbiological results in laboratory systems; antimicrobial consumption in pharmacy databases; vaccination in registries; animal infections in veterinary networks; and climate, pollution, wastewater, and vector data in other agencies. A digitally enabled One Health model wouldn't indiscriminately centralize everything. It would set up interoperable, privacy-preserving pathways that allow relevant signals to be combined across space and time.

Such infrastructure could detect an unusual pediatric syndrome, connect it with a veterinary or environmental alert, map it against weather and mobility patterns, and quickly return guidance to clinicians. Wastewater and environmental sequencing could complement clinical testing, while genomic epidemiology could distinguish community transmission from healthcare-associated clusters. Federated analysis could let institutions collaborate without transferring identifiable child-level data. Dashboards could provide age-stratified incidence and resistance patterns, and automated alerts could support—rather than replace—public health judgment. This direction aligns with the WHO global strategy on digital health, but pediatric implementation needs explicit age-sensitive priorities (6).

AI may enhance forecasting, image and signal interpretation, triage, diagnostic support, and antimicrobial decision-making. Still, the gap between model development and clinical benefit remains wide. A systematic review of predictive AI implemented in pediatric practice found few real-world implementations and inconsistent evaluation of clinical, workflow, and human outcomes (7). In pediatric infectious diseases, digital technologies show promise for monitoring, access, communication, and timely intervention, but infrastructure and implementation gaps persist (8). Future research has to move beyond retrospective accuracy. Models should be externally validated across ages, settings, ethnic and socioeconomic groups; compared with existing care; evaluated prospectively; monitored for performance drift; and designed with clinicians, families, and public health professionals.

## From faster diagnosis to smarter stewardship

Digital transformation can be most immediately useful where uncertainty drives unnecessary treatment. Rapid molecular tests, multiplex platforms, biomarkers, and metagenomic next-generation sequencing can shorten diagnostic pathways and reveal uncommon or mixed infections. Recent pediatric evidence suggests that metagenomic sequencing offers high sensitivity and frequently changes antimicrobial management, although cost, interpretation, standardization, and contamination remain challenges (8). Diagnostic innovation therefore has to be coupled to diagnostic stewardship: selecting the right test, for the right child, at the right time, and interpreting it in its clinical and epidemiological context.

Antimicrobial stewardship should likewise evolve from periodic prescription review to a continuously learning One Health function. Clinical decision support could integrate age, syndrome, allergy history, previous microbiology, local resistance, drug availability, pharmacokinetics, and organ function. At population level, pediatric prescribing could be analyzed alongside resistance and antimicrobial use in animals and, when feasible, environmental reservoirs. European integrated analyses already demonstrate the importance of coordinated surveillance across human and food-producing animal sectors and call for inclusion of environmental data (9). The next step is making this information actionable at the bedside without increasing alert fatigue or inequity.

The same approach applies to healthcare-associated infections. Neonatal and pediatric intensive care, oncology, transplantation, surgery, and long-term technology dependence create distinctive risks. Electronic surveillance can combine device days, microbiology, antibiotic exposure, staffing, movement through wards, and environmental sampling to identify preventable harm earlier. Genomic data can clarify transmission, while real-time feedback can improve adherence to infection-prevention bundles. These tools must be judged by reduced infections, antimicrobial use, length of stay, and family burden—not merely by technical detection rates.

## A research agenda linking the microbiome, exposome, and infection

One Health and digital transformation also open up a chance to study mechanisms rather than correlations alone. Longitudinal pediatric cohorts can combine clinical phenotypes with gut and respiratory microbiome profiles, pathogen detection, antimicrobial exposure, diet, vaccination, air quality, temperature, housing, green space, and social determinants. Wearable and geospatial technologies may improve exposure measurement, while multi-omics and causal inference can help identify pathways connecting pollution or climate-related exposures to mucosal immunity and infection.

This agenda requires restraint as well as ambition. Microbiome signatures can be population- and context-specific; sequencing detects nucleic acid rather than necessarily viable disease-causing organisms; environmental measurements are prone to misclassification; and highly dimensional models can generate convincing but non-reproducible associations. Studies should therefore prioritize standardized sampling, transparent analytic plans, clinically meaningful endpoints, diverse populations, and independent validation. Interventions—such as reducing harmful environmental exposure, improving ventilation, optimizing antibiotic use, or restoring microbial ecosystems—should ultimately be tested for their ability to improve child health.

## Emerging infections and the global equity challenge

Emerging and re-emerging infections are a defining challenge for pediatric infectious diseases. Zoonotic spillover, vector expansion, climate disruption, conflict, displacement, declining vaccine coverage, and fragile health systems can transform a local signal into a regional or global emergency. Children may be disproportionately affected yet remain under-represented in surveillance, therapeutic trials, and emergency preparedness. Readiness therefore requires pediatric protocols, pre-approved adaptive trial platforms, biobanking and genomic capacity, rapid data sharing, and pathways that can pivot from endemic diseases to outbreaks without abandoning routine care.

Preparedness can't be separated from access. Infectious diseases remain leading causes of childhood death, while many low- and middle-income countries (LMICs) face limited access to laboratories, imaging, oxygen, vaccines, child-appropriate formulations, and effective antimicrobials (10). Innovations developed during emergencies have too often reached the populations bearing the greatest burden late, at unaffordable prices, or not at all. Equity must be designed across the full pathway—from priority setting and product development to procurement, delivery, and post-implementation evaluation. Digital tools should function with intermittent connectivity, limited computing resources, and local languages rather than widening the digital divide.

Research in LMICs must also move beyond extractive models. Institutions and investigators in affected countries should lead agenda setting, governance, analysis, authorship, and translation into policy; communities should participate from protocol design onward; and data and biological samples should be governed through fair, transparent agreements. Sustainable investment in laboratories, ethics and regulatory systems, trial networks, data stewardship, and research careers is itself a preparedness intervention. WHO guidance calls for country-led, continuously functional clinical-trial ecosystems and highlights the persistent under-representation of children (11). Pediatric innovation should be judged not only by discovery, but by whether affordable diagnostics, vaccines, medicines, and evidence reach every child who needs them.

## Governance, equity, and the pediatric digital compact

The greatest risk is that digital One Health becomes a technically sophisticated system that doesn't serve the children at highest risk. Data gaps aren't random: populations with limited healthcare access, unstable housing, migration, conflict, or weak connectivity are often least visible to digital systems. Algorithms trained on data from tertiary hospitals or high-income settings may underperform elsewhere. Surveillance may also create harms through stigmatization of communities, locations, occupations, or animal contacts.

Children merit heightened safeguards because they can't always provide consent, their data may remain identifiable across a lifetime, and decisions made today can shape future opportunities. WHO guidance emphasizes that AI for health must be governed by ethics, human rights, transparency, accountability, and protection of autonomy (12). Pediatric systems should apply data minimization, clear purpose limitation, age-appropriate assent, meaningful caregiver engagement, cybersecurity, auditability, and defined human oversight. Families and young people should participate in determining which uses of data are acceptable and valuable.

Equity must be an outcome, not a generic principle. Every digital intervention should report who was included, who was excluded, who benefited, and who carried additional burdens. Open standards, adaptable low-resource tools, multilingual communication, and capacity building are essential. Investment is also needed in a workforce fluent across pediatrics, infectious diseases, microbiology, epidemiology, veterinary and environmental sciences, data science, behavioral science, ethics, and implementation research. No single profession can deliver this transformation.

## Five commitments for the specialty

The pediatric infectious diseases community should make five commitments. First, we should build interoperable, age-stratified One Health surveillance that connects clinical, laboratory, genomic, antimicrobial, veterinary, vector, wastewater, climate, and pollution signals. Second, we should evaluate digital tools by prospective clinical and public health impact, usability, sustainability, and equity—not by algorithmic performance alone. Third, we should integrate diagnostic stewardship, antimicrobial stewardship, and infection prevention into the same learning system. Fourth, we should establish longitudinal research platforms linking the microbiome and exposome to infection while moving toward testable preventive interventions. Fifth, we should create a pediatric digital governance framework in which children's rights, family participation, transparency, and accountability are designed in from the start.

These commitments also define a broad research scope for the specialty: emerging and vector-borne infections; zoonoses and foodborne disease; AMR; healthcare-associated infections; vaccines; microbiome science; environmental determinants; digital diagnostics; AI; implementation; and health equity. The unifying question is whether research can connect these domains in ways that improve decisions for children.

## Conclusion

The future of pediatric infectious diseases will be determined not only by which pathogens emerge, but by whether we can recognize and respond to connected threats before they become crises. One Health supplies the necessary systems perspective; digital transformation provides tools to make that perspective operational. Neither is sufficient alone. One Health without interoperable data risks remaining rhetorical, while digital innovation without ecological context, clinical validation, and ethical governance risks producing faster fragmentation.

Our grand challenge is to create a trustworthy, equitable learning ecosystem in which information flows responsibly from the child's bedside to the laboratory, community, environment, and public health system—and back again as timely action. Pediatric infectious disease specialists should help lead this transition because we understand both the urgency of infection and the long horizon of childhood. Protecting children today also protects the microbial, social, and environmental conditions in which future generations will live.
